# Fungal-bacterial diversity and microbiome complexity predict ecosystem functioning

**DOI:** 10.1038/s41467-019-12798-y

**Published:** 2019-10-24

**Authors:** Cameron Wagg, Klaus Schlaeppi, Samiran Banerjee, Eiko E. Kuramae, Marcel G. A. van der Heijden

**Affiliations:** 1 Plant Soil Interactions, Division Agroecology and Environment, Agroscope, Reckenholzstrasse 191, CH-8046 Zürich, Switzerland; 2 Department of Evolutionary Biology and Environmental Studies, University of Zürich, Winterthurerstrasse 190, Zürich, CH-8057 Switzerland; 3 Fredericton Research and Development Center, Agriculture and Agri-Food Canada, 850 Lincoln Rd, Fredericton, NB E3B 4Z7 Canada; 4 Institute of Plant Sciences, University of Bern, Altenbergrain 21, CH-3013 Bern, Switzerland; 5 Department of Microbial Ecology, Netherlands Institute of Ecology (NIOO-KNAW), 6708 PB Wageningen, The Netherlands; 6 Department of Plant and Microbial Biology, University of Zurich, Zollikerstrasse 107, CH-8008 Zurich, Switzerland

**Keywords:** Microbial ecology, Ecosystem ecology

## Abstract

The soil microbiome is highly diverse and comprises up to one quarter of Earth’s diversity. Yet, how such a diverse and functionally complex microbiome influences ecosystem functioning remains unclear. Here we manipulated the soil microbiome in experimental grassland ecosystems and observed that microbiome diversity and microbial network complexity positively influenced multiple ecosystem functions related to nutrient cycling (e.g. multifunctionality). Grassland microcosms with poorly developed microbial networks and reduced microbial richness had the lowest multifunctionality due to fewer taxa present that support the same function (redundancy) and lower diversity of taxa that support different functions (reduced  functional uniqueness). Moreover, different microbial taxa explained different ecosystem functions pointing to the significance of functional diversity in microbial communities. These findings indicate the importance of microbial interactions within and among fungal and bacterial communities for enhancing ecosystem performance and demonstrate that the extinction of complex ecological associations belowground can impair ecosystem functioning.

## Abstract (web summary)

There is ongoing interest in linking soil microbial diversity to ecosystem function. Here the authors manipulate the diversity and composition of microbial communities and show that complex microbial networks contribute more to ecosystem multifunctionality than simpler or low-diversity networks.

## Introduction

Microbes are the unseen majority on Earth and comprise a large portion of life’s genetic diversity^1–3^. A multitude of microorganisms associate with humans, animals, insects, plants, and soils around the globe^4–8^. In each of these biomes, microbes usually form highly diverse and complex communities that collectively function as a microbiome. Earlier studies focused on the description of these microbial communities, but currently there is much interest to link microbiome composition and diversity to function^1,9,10^. This is not surprising because it is well known that microbes impact all living organisms and play a central role in many biogeochemical cycles on earth, driving global carbon and nutrient cycling with direct feedback effects on ecosystem functioning and productivity^1–3^.

Experiments carried out in microcosms^11–17^ and at global observational scales^18,19^ revealed that microbial diversity is linked to ecosystem functioning, implying that communities with higher microbial richness perform better. The extremely high microbial diversity on small spatial scales has led to hypotheses that these highly diverse microbiomes are functionally redundant^20^. Yet, functional redundancy is an important feature of biodiversity as greater diversity provides a greater likelihood that some species are present that can perform a function under temporally and spatially varying conditions and buffers functioning against the loss taxa so that ecosystem functioning is maintained^21–23^. Furthermore, although such a vast soil microbial diversity may appear to be functionally redundant, microbes are involved in multiple functions simultaneously and thus functional redundancy is likely to fade as more functions are considered, as has been shown for plant richness–multifunctionaliy relationships^24,25^. To understand how changes in soil biodiversity affect ecosystem functioning it is therefore important to consider not only whether the total number of taxa present relates to a function, but how the reduction in the number of species that support a single function relates to the loss of multiple functions simultaneously.

Importantly the influence of an individual species on an ecosystem function is not independent of other species present and is a result of a myriad of positive and negative, direct and indirect associations among the different species that as a whole drive ecosystem functioning. For instance, microbial communities are not only characterized by the number and composition of taxa, but also by the ecological associations among microbiome members. In recent years, microbial co-occurrence analyses have shed light on microbiome complexity and the interrelationships among community members^26^. Emerging studies have revealed that microbiomes are structured, and form complex interconnected microbial networks^26–31^, where microbes associate with each other directly or indirectly through processes, such as competition, facilitation, and inhibition. The complexity of these microbial networks and its relation to function is not necessarily determined by the number of taxa in the community, but rather by the number of associations that those taxa share amongst them^31^. A next frontier is now to empirically test whether changes in microbiome complexity, as indicated by both the diversity and interconnectivity among co-occurring microbes, is important for the way microbial communities affect ecosystem functioning.

By fractionating soil organisms according to size, using filters of decreasing mesh size we have previously shown that the loss of soil biodiversity resulted in reduced plant diversity, productivity, nutrient retention, and belowground carbon allocation using self-contained grassland microcosms that restrict external contamination^15^. However, the role of microbiome diversity, functional redundancy, and network complexity within and among bacterial and fungal communities in regulating ecosystem performance has not been assessed along such a soil biodiversity gradient. Thus, we capitalize on this model system with a strong gradient in soil biodiversity here to further assess these different features of soil microbial diversity and their relationship with 10 soil functions that are known to be mediated by soil microbes^1–3^, and that reflect nutrient cycling efficiency, here termed soil multifunctionality^25,32^. We used soil collected from these microcosms and used next generation sequencing to characterize the fungal and bacterial soil microbiome (see the “Methods” section).

Although next generation sequencing tools have allowed us to capture a vast diversity of soil microbes, many of the taxa detected may not play a significant role in the ecosystem functions of interest, thus resulting in ‘noise’ that may obscure the realized diversity–function relationship. This is in contrast to classic plant diversity–productivity relationship where each plant present inherently contributes biomass to the net ecosystem productivity. Thus, we used feature selection, a statistical tool, to identify taxa that contribute to predicting the performance of each ecosystem function considered (see the “Methods” section^33^). This provided us with the identities of fungal and bacterial taxa that support a function (directly or indirectly), thereby removing such ‘noise’ in assessing diversity–function relationships. The association of microbial taxa to functions then allowed us to further assess the effects of greater microbiome diversity on increasing the redundancy of taxa that support a common function, where greater redundancy means that there are a greater number of taxa that support the same function. We also quantified the functional diversity within the microbial communities using the functional uniqueness index, which is the product of Raos quadradic entropy and the inverse Simpsons index and summarizes the diversity in the relative abundance among microbes that support different functions^34^.

Here we hypothesize (1) that microbiome richness and microbial network complexity promotes ecosystem multifunctionality, (*2*) that if a given ecosystem function is not the result of the presence of a single taxon, then having more taxa present that positively contribute, directly or indirectly, to the underlying processes that drive a response in a function should lead to a positive redundancy–function relationship. At the same time, if greater microbial richness enhances ecosystem multifunctionality then it would be hypothesized that this is because (3) greater richness provides a greater diversity of taxa that support multiple different functions resulting in a positive functional uniqueness–ecosystem multifunctionality relationship. We assessed microbiome complexity by first generating a meta-association matrix including all fungal and bacterial taxa from all microcosms. We used a cross-validation and a graphical model inference framework to define the most parsimonious links among the taxa^35^ (see the “Methods” section). From this, sub-networks based on taxa present in specific microcosms were used to generate indices of soil microbiome complexity (linkage density) among fungal and bacterial taxa.

This work demonstrates that more complex microbial networks contribute more to improved ecosystem function multifunctionality than simpler or low-diversity networks. Moreover, different microbes support different functions pointing to the significance of functional diversity within microbial communities.

## Results

### Microbial network complexity and ecosystem functioning

**Fig. 1:**
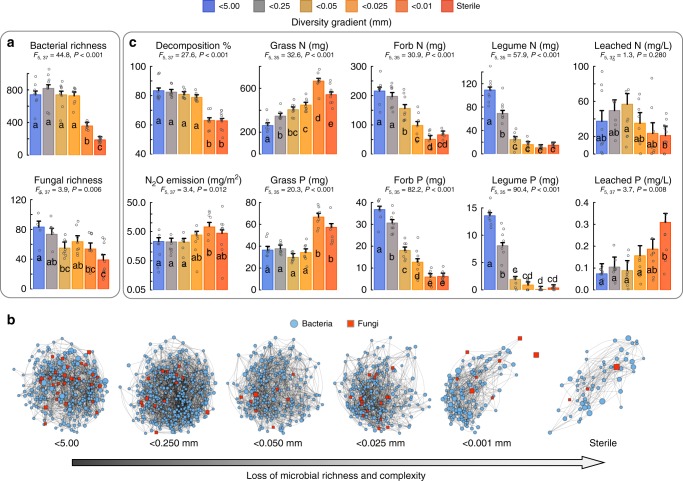
Soil microbial community composition and ecosystem functioning with progressive simplification of the soil biome. The soil diversity gradient was established by filtering inoculum through different meshes: ≤5; ≤0.25; ≤0.05, ≤0.025, ≤0.001 mm, or adding sterilized soil inoculum. Shown are (**a**) the mean richness of bacterial and fungal OTUs and (**b**) the microbial association networks where blue circles indicate individual bacterial operational taxonomic units (OTUs), red square nodes indicate individual fungal OTUs and lines indicate interlinkage between OTUs. For visual clarity, only OTUs that were detected to be present in 75% of all replicates within each treatment level are illustrated. Networks are based on subsets of a meta-network matrix where subset matrices were generated using only OTUs present within a treatment. Connectedness within the meta-network was 2.2% (70,830 links out of 3,290,596 possible links). Larger nodes indicate the OTU was relatively more abundant within that particular treatment. **c** The mean of ecosystem functions related to nutrient cycling for each of the soil community treatments. Error bars in **a** and **c** are standard errors and different letters indicate significant differences (Tukey HSD) between treatments (*n* = 8 for each treatment level with *n* = 10 for the sterile treatment, except for plant nutrients where *n* = 8)

**Fig. 2:**
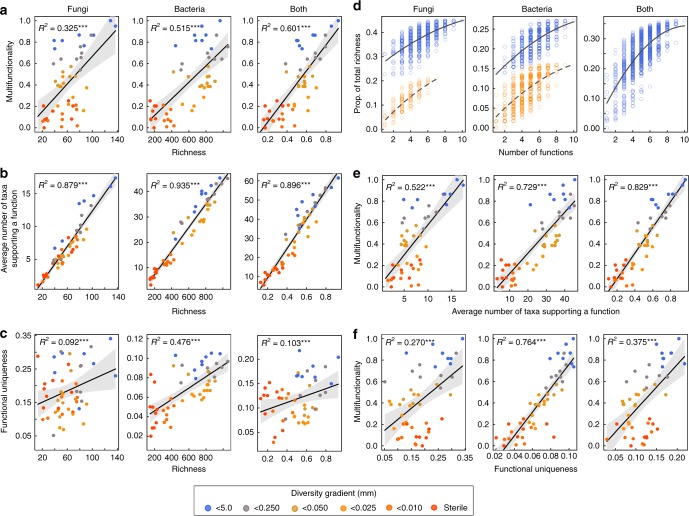
Relationship between microbial composition indices and soil multifunctionality. The total fungal and bacterial OTU richness are shown in relation to **a** multifunctionality, **b** the average number of taxa that support a function, where larger values indicate that on average there are more taxa that support the same function, and **c** functional uniqueness where larger values indicate a greater dissimilarity among taxa in the functions they support. **d** Proportion of the microbial community needed to support a given combination of functions, from single functions to all 10 functions, illustrating that more taxa are needed to support multiple functions as more functions are considered^25^. Dashed regression lines show combinations of functions (in orange) that exclude P uptake by legumes and forbs and N uptake by legumes for fungi and N_2_O emissions for bacteria. These cluster out because these functions had a relatively high number of taxa that supported them (Table S5). Thus, there is a smaller proportion of the community that supports individual functions when these are excluded. Ecosystem multifunctionality was positively related to (**e**) the average number of taxa that support a function and (**f**) functional uniqueness. For **a**–**c** both fungi and bacterial richness was combined by taking their scaled (between 0 and 1) average richness. Significance is indicated by *P < 0.05, **P < 0.01, ***P < 0.001 for each linear regression

The filtering of soil organisms along the diversity gradient had a strong effect on reducing microbiome richness and the association network complexity (linkage density among those taxa) (Fig. 1a, b, Supplementary Fig. 1 and Supplementary Tables 1 and 2). Soil microbial network complexity declined strongly along the gradient with the highest richness and microbial network connectivity in microcosms receiving inoculum passing the 5 mm filter and lowest richness and connectivity in the sterile control treatment (Fig. 1a, b, and Supplementary Tables 1 and 2). The decomposition of plant litter and nutrient (N and P) uptake by forbs and legumes declined over our soil diversity gradient, while the emission of the greenhouse gas N_2_O and P leaching (two soil functions which lead to nutrient losses and thus impair ecosystem functioning) decreased at higher levels of microbiome richness and complexity along the diversity gradient. Nutrient uptake by grasses increased with a reduction of microbiome richness and complexity along the soil diversity gradient (Fig. 1c). All ecosystem functions were significantly related to both bacterial and fungal richness along the soil diversity gradient (Supplementary Fig. 2) and thus, fungal and bacterial richness was also positively related to ecosystem multifunctionality (Fig. 2a). By fitting the diversity gradient treatment levels after first explaining the relationships with microbiome richness relationships, we found that there was significant variation among the treatments for the majority of ecosystem functions (Supplementary Table 3), suggesting that these ecosystem functions are regulated by additional changes in the soil biome among treatments not captured by the changes in microbial richness alone (see below).

### Functional diversity in microbial communities

By identifying suites of taxa that together support functions, we found that 27.1% of bacterial taxa and 44.9% of the fungal taxa were associated with supporting at least one ecosystem function (i.e. have coefficients related to increasing plant nutrient uptake and litter decomposition or reducing nutrient losses). Much fewer taxa were found to inhibit functions and of the bacterial and fungal taxa, 14.4% and 19.2% were negatively associated with a function (Supplementary Fig. 3 and see Supplementary Table 4 for taxonomic details). Here we focus on the taxa that support a given function but results for those that contribute negatively to functioning are presented in the Supplementary Materials (Supplementary Tables 1, 2, and 4). The number of microbial taxa that supported ecosystem functioning (i.e. have coefficients related to greater plant nutrient uptake and litter decomposition or lower N_2_O emissions and nutrient leaching) declined along our soil diversity gradient (Supplementary Tables 1 and 2). Many of the taxa that were related to functions were only associated with one or a few functions (Supplementary Fig. 3) and functions were rarely supported by a similar composition of taxa, with the exception of N and P uptake by legumes (51% overlap) and by grasses (55% overlap) (Supplementary Table 5). Greater richness of fungal and bacterial taxa also resulted in a greater number of taxa that support a function (Fig. 2b). Moreover, greater richness of bacterial and fungal taxa was also associated to greater functional uniqueness within the microbial community indicating a greater diversity in the relative abundance of microbes that support different functions (Fig. 2c).

As would be expected, all individual functions were better predicted by the presence of several taxa that supported the same function (greater redundancy), with the exception of N leaching, where the abundance of a single fungal taxa best predicted reduced N leaching (see Supplementary Fig. 4, Table S5). This suggests N leaching was loosely associated with changes in microbial composition in our system since previously we noted that N leaching was related to the performance of grasses and their ability to capture nitrate in this system^15^. Nonetheless, by considering more ecosystem functions, more fungal and bacterial taxa were required to support multiple functions simultaneously (Fig. 2d) as has been previously demonstrated in diverse plant communities where more plant species are required to support more functions^25^. Moreover, by increasing fungal and bacterial richness greater multifunctionality was supported because of an increase in the number of taxa that support a function (Fig. 2e), as well as an increase in the functional uniqueness within the fungal and bacterial communities (Fig. 2f). Taken altogether these results demonstrate the critical importance of soil microbiome diversity as it shows that a more rich and diverse microbiome provides a greater likelihood that (1) there will be more taxa present that are needed to support any given function, which is in support of the diversity–redundancy hypotheses^21–23^ and (*2*) greater richness provides a greater number of taxa that support different functions in support of the diversity–multifunctionality hypothesis previously observed along plant diversity gradients^24,25^.

### Functional complexity in microbial communities

**Fig. 3:**
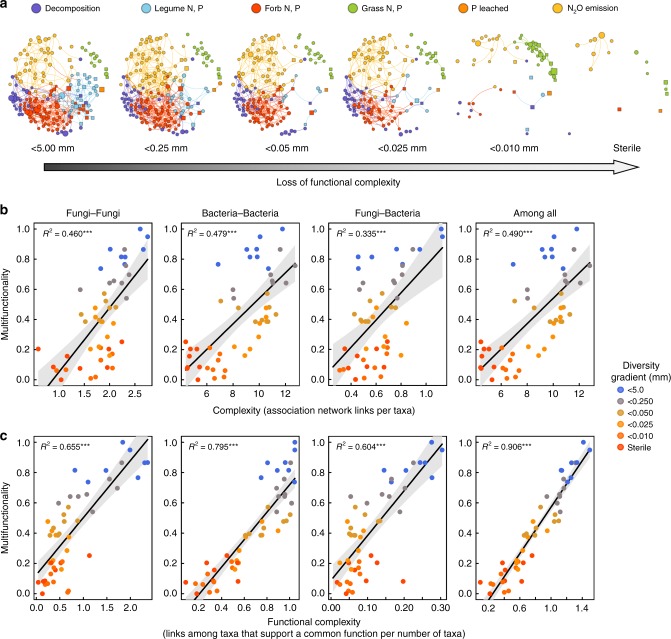
Relationship between microbial association network complexity and multifunctionality. **a** Illustrates OTUs that were detected to predict a common ecosystem function (i.e. taxa with coefficients that are related to increased plant nutrient uptake and decomposition or reduce nutrient loss). Nodes (OTUs) and links are colored by the functions with which they are most strongly associated (Note: ‘functional complexity’ is defined as the ratio of the total number of links). Node size indicates the OTU was relatively more abundant within that particular treatment. For visual clarity, only OTUs that were detected to be present in 75% of all replicates of each treatment level are illustrated. Shown in (**b**) are the relationships between multifunctionality and the overall association network complexity (shown in Fig. 1b) among fungi–fungi, bacteria–bacteria, fung–bacteria and among all fungi and bacteria. The same is shown in (**c**) except only considering the links among taxa that support a function (shown in **a**). Significance is indicated by *P < 0.05, **P < 0.01, ***P < 0.001 for each linear regression. Results after detrending for richness are shown in Table 1

By combing results that identify combinations of taxa that support each function with the results from the association network among all taxa we were able to define a ‘functional complexity’ index, which is the linkage density among taxa that also contributed to predicting greater ecosystem function. As with all other indices of microbial diversity, functional complexity also decreased along the soil diversity gradient (Fig. 3a, Supplementary Tables 1 and 2) and the overall microbial complexity in the microbial association network was strongly positively related with multifunctionality (Fig. 3b). This is because greater network complexity is often dependent upon the number of nodes (taxa) within the network, however, a greater network complexity alone did not always provide a better explanation of multifunctionality than the overall microbial richness detected (see Fig. 2a for comparison). By combining information on the identity of the taxa that support each function with that of the association network we found that greater complexity among fungal and bacterial communities that support a common function increased plant nutrient uptake and litter decomposition, as well as reduced nutrient losses from N_2_O emissions, but showed weak to no relationships with nutrient leaching (Supplementary Figs. 5 and 6). Nonetheless by considering the network complexity among taxa that support all functions provided we obtained the strongest relationships with multifunctionality (Fig. 3c). Since network complexity can be dependent upon richness, we also assessed whether overall microbial richness could explain the strong relationship between functional network complexity and soil multifunctionality. We found that by first detrending for richness, we could explain a highly significant proportion of the relationship but that the residual effect of functional complexity on multifunctionality also remained highly significant even after accounting for microbial richness effects (Table 1 and Supplementary Table 2).

**Table 1:** Effects of soil microbial network characteristics on ecosystem multifunctionality

	SES	SE	*P*	*R* ^2^
*Complexity*				
Bact–Bact	0.716	0.110	<0.001	0.479
Fungi–Fungi	0.696	0.111	<0.001	0.460
Bact–Fungi	0.592	0.123	<0.001	0.336
Among all	0.723	0.109	<0.001	0.490
*Funct. comp*.				
Bact–Bact	0.911	0.068	<0.001	0.795
Fungi–Fungi	0.805	0.086	<0.001	0.655
Bact–Fungi	0.777	0.093	<0.001	0.604
Among all	0.969	0.046	<0.001	0.906
*Complexity after detrending for microbial richness* ^a^				
Bact–Bact	−0.308	0.148	0.044	0.086
Fungi–Fungi	0.343	0.137	0.016	0.120
Bact–Fungi	0.113	0.144	0.438	0.013
Among all	−0.238	0.151	0.122	0.051
*Funct. complexity after detrending for microbial richness.* ^a^				
Bact–Bact	0.507	0.125	<0.001	0.263
Fungi–Fungi	0.395	0.105	<0.001	0.234
Bact–Fungi	0.513	0.124	<0.001	0.271
Among all	0.543	0.121	<0.001	0.306

*Note*: Standardized effect (SES) associated standard error (SE) and *R*^2^ are provided for each network characteristic (regression)

^a^Results are for effects of soil microbial community characteristics on ecosystem multifunctionality after de-trending for richness

Intriguingly our results show that although soil multifunctionality was strongly related to all soil microbiome diversity characteristics in all cases, indices that considered both fungal and bacterial communities together were generally more predictive of soil multifunctionality than those considering only one of the two (see Figs. 2, 3 and Table 1). This points to the importance of the diversity in both the fungal and bacterial communities for supporting multiple ecosystem functions. Furthermore, we fit each community characteristic in order by which they were derived (total richness, number of taxa that support a function, functional uniqueness, and the network complexity among taxa that support a function) to see how each additional characteristic contributed to explaining ecosystem multifunctionality. For the most part each community characteristic was able to capture an additional significant proportion of variation in multifunctionality, with the exception of functional uniqueness when both fungal and bacterial communities were considered (Table 2). Although here we focus on the soil microbiome our filtering treatment gradient also likely affected a number of other soil fauna including nematodes and protozoa that are not assessed here. To capture this unknown effect of filtering our organisms based on size we also fit the treatment gradient (factor) to see how much residual variation among treatments remains after first explaining away all other microbial community characteristics. In all cases we found that there was a significant amount of variation among treatments remaining even after accounting for the multiple microbial community characteristics quantified here (Table 2). This indicates that although microbial richness, functional redundancy, diversity, and complexity are all strongly related to maintaining multiple ecosystem functions that comprise nutrient cycling, there is still a significant amount that remains unexplained that is likely to changes in other soil fauna not quantified in our study.

**Table 2:** The percent of variation (*%*SS) in multifunctionality that is explained by each community characteristic

	%SS	*P*
*Bacteria*		
Richness	54.72	<0.001
Ave. # supporting taxa	32.69	<0.001
Uniqueness	1.15	0.003
Functional complexity	2.40	<0.001
Treatment gradient	3.53	<0.001
*Fungi*		
Richness	33.30	<0.001
Ave. # supporting taxa	28.71	<0.001
Uniqueness	2.10	0.001
Functional complexity	3.03	<0.001
Treatment gradient	26.04	<0.001
*Both*		
Richness	60.79	<0.001
Ave. # supporting taxa	31.28	<0.001
Uniqueness	0.03	0.499
Functional complexity	0.89	0.003
Treatment gradient	2.42	<0.001

*Note*: Results show the bacteria only, fungi only, and both combined. The %SS is the sequential sum of squares (type I, ANOVA) expressed as a percentage of the total and *P* indicates the significance. The %SS for block and residuals are not shown and are represented by the %SS needed to achieve 100%

## Discussion

Soils harbor a vast diversity of microbes^1–3^ and recent studies have identified the drivers of microbiome composition, network association patterns, and complexity in a wide range of ecosystems^1,6–8,26,27^. However, there is a pressing need for moving beyond mere descriptions of microbial community composition and delving into the functional implication of compositional patterns and changes in microbial network structure as was highlighted recently^1,10,11^. In particular, while a large number of studies employing microbial network analysis have enriched our understanding of microbial co-occurrence patterns in various soil ecosystems^26–31^, very little is known of whether differences in the structure of microbial networks have consequences for microbiome functioning. Although earlier social network studies have linked network structure to functional complexity^36,37^, the task of relating microbial community structure to function is a non-trivial one largely due to the contentious nature of structure–function relationship that has perplexed microbial ecologists for the last two decades^38–41^. To our knowledge, this is one of the first studies to link microbial network complexity to ecosystem multifunctionality. Our results reveal that while taxonomic richness is an important feature that drives multifunctionality it does so because richness supports greater microbiome complexity and interkingdom associations (here by considering fungi and bacteria simultaneously). Thus, combining these microbiome characteristics can enhance our assessment of the attributes of soil microbiome diversity that explain an aggregate of process functions, i.e., soil multifunctionality. This adds a new dimension to earlier observations that soil biodiversity and microbial richness act as a driver of soil multifunctionality^18,19^.

This study also shows that the impact of microbial communities and their taxonomic richness on ecosystem functioning may be better understood by considering various microbiome characteristics that may include: a) functional redundancy—here the increasing number of taxa that support a common function, b) the diversity of taxa that support different functions and c) the complexity of associations among taxa that support functioning. This is because here we show that greater microbiome richness is needed to support (1) greater functional redundancy to secure individual functions and (2) greater functional diversity to support multiple functions simultaneously.

There are a number of experimental studies which manipulated the diversity of plant^24,25,42–44^ and microbial communities^11–16^ that have demonstrated the importance of biodiversity for ecosystem functioning, and for maintaining multiple ecosystem functions. Experiments in plant communities have provided empirical evidence for the widely regarded insurance and redundancy hypotheses of biodiversity for sustaining ecosystem functioning, where greater richness can provide a greater guarantee of the maintenance in functioning under various spatial–temporal environmental conditions^22–25^. For soil microbial communities confronted with a potentially large functional redundancy^20^, here we similarly show that greater soil microbiome diversity can also ensure the greater performance in multiple ecosystem functions. This was largely due to the effects of greater microbiome richness increasing (1) the redundancy effect of having more taxa present that support the same function and (2) an increase in the presence of microbes that were for the most part associated with different ecosystem functions. These results show the importance of maintaining a greater taxonomic richness because it supports greater functional redundancy and diversity that parallels observations in aboveground plant communities^21–25,42–44^. Importantly, functional redundancy and diversity are both key features of biodiversity that provide support for the ‘insurance’^21,22^ and ‘rivit-redundancy’^21,45^ hypotheses and the ‘portfolio’ effect^46^ as to why greater biodiversity is needed to maintain greater functioning. Our findings further extend these concepts to show that greater microbial richness also provides greater association complexity within microbial communities and resultantly a greater association among taxa that support the multiple functions of interest. Further we found that by combining results from both fungal and bacterial communities on their functional associations, along with results from network analyses, we could achieve some of the strongest relationship with multifunctionality. This supports our hypothesis (3) that a more taxonomically rich soil microbiome can underpin soil multifunctionality because it also ensures greater association complexity among microbes that together are required to support multiple ecosystem functions simultaneously.

Perhaps what is most intriguing about our findings is that often the consideration of both fungi and bacteria together improved our ability to predict soil multifunctionality. Until now, most microbiome studies have focused on particular groups (e.g. bacteria or fungi), while still relatively few assessed other key groups of soil organisms, such as protists, Archaea, and nematodes^29,47,48^ in order to obtain a more complete picture of the soil biome. Here we found that considering both fungal and bacterial community characteristics simultaneously was often a better predictor of multifunctionality in nutrient cycling compared to considering these two microbial kingdoms separately. This is in line with earlier observations revealing that there is division of metabolic labor among microbes leading to complementarity among those with unique physiological properties, such as between fungi and bacteria^49,50^. For instance, litter decomposition may be performed by distinct groups of soil microbes that inhabit different parts of the soil horizon^51^. Moreover, it has been shown that different plant symbionts (arbuscular mycorrhizal fungi and nitrogen-fixing bacteria) can complement each other by providing different limiting nutrients to plants resulting in higher plant productivity^52^. This points to the importance of microbial interkingdom associations as a driver of ecosystem functioning and parallels recent observations that associations among guilds of microbes promote plant health in the model plant *Arabidopsis thaliana*^53^. Such unseen synergisms might be much more widespread and ecologically important for the soil microbiome functioning than previously thought.

Although here our focus was solely on the soil microbiome encompassing soil fungi and bacteria, our results also allude that including information from other organismal groups beyond fungi and bacteria may further improve our ability to predict soil multifunctionality. Therefore, a next challenge is to link the composition of the soil biome, including multitrophic levels, to ecosystem functioning. It has been hypothesized that vertical diversity (among guilds of organisms) may be just as important, if not more, than the horizontal diversity within a single guild of organisms^54^. Considering this, it is important to note that the filtering approach that we used to manipulate microbial communities also altered the composition of other key groups of soil biota not assessed in our study. These unassessed groups, such as microbial and fungal feeders, may also have contributed to the observed effects directly or indirectly and this deserves further attention in future work (see Table 2). Although here we focus on the soil microbiome, there remains numerous facilitative, antagonistic, and multi-trophic interactions among the many individual members of the soil biome that are still poorly understood as to how such vertical diversity affects soil multifunctionality that needs further exploration.

In nature, soil ecosystems are highly heterogeneous since soil microbial biodiversity hot spots can form spatial and temporally within soil aggregates^55–57^ and microbial abundance and diversity declining with greater soil depth^58,59^. This spatial heterogeneity likely plays an important role for the interactions among microbes and the mechanisms by which more complex and diverse communities drive various nutrient cycling processes on small spatial scales. For instance, fungal hyphal networks can span air pores within the soil facilitating the movement of bacterial communities to new resource patches^49^. Furthermore, diverse microbial interactions within soil aggregates are likely not only microbial diversity hot spots but are likely also hot spots for key soil processes^57^. Considering these additional spatial complexities of the soil ecosystem in natural environments together with our results could indicate the further importance of various indices of microbial diversity and functional complexity across spatial and temporal scales as has been shown for aboveground plant communities^24^. Moreover, perturbations to the soil ecosystem through compaction and tilling that physically damage larger soil organisms^60^ and restrict movement among soil pore space^49,61^, will likely not only result in the loss of soil microbial diversity and the structural spatial complexities in natural soil environments, but also its ability to function and cycle nutrients between above and belowground compartments effectively. Considering this, our approach of using a model system with a relatively homogenous soil environment may have underestimate the importance of the complexity of soil microbial diversity and its role in supporting ecosystem function that requires further investigation in situ.

Recent studies have shown that microbial network complexity varies between different ecosystem types. For instance, network complexity was much higher in late successional fields compared to early successional fields^29^. Moreover, organically managed agricultural fields harbored much more complex fungal networks with many more keystone taxa, compared to conventional managed fields^31^. Extrapolation of the findings in this study, to these systems, implies that the microbial contribution to ecosystem functioning is higher in systems with higher association complexity, such as in late successional fields and the organically managed fields^29–31^. Our study emphasizes that both horizontal functional diversity (i.e. diversity within a guild of organisms such as fungi) and vertical or interkingdom functional diversity (e.g. diversity among functional guilds, such as among fungi and bacteria) are both important for maintaining ecosystem functioning. Our work further demonstrates that the extinction of complex ecological interactions belowground impairs important ecosystem services that soils provide us.

## Methods

### Microcosms

The plant community and soil communities were established within self-contained microcosms with air and water entering the microcosm through purifying filters (0.2 µm in pore size) to prevent microbial contamination from the outside and maintain a broad gradient of soil community complexities. A detailed description of the microcosms and the experimental set-up is provided in earlier work^15,62^. Briefly, each microcosm was filled with 5 kg of a standardized 1:1 sand:field soil mix, which was previously sterilized. Soil communities were created using 250 g of soil collected from a long-term grassland and then sieved through a series of decreasing mesh size: 5, 0.25, 0.05, 0.025, 0.01 mm or sterile. Soil material not passing through the sieve was sterilized by autoclaving and was added to the microcosm substrate along with the living fraction passing through the sieves. The total amount of inoculum (250 g soil) added to each microcosm represented <5% of the total soil volume and it had negligible effects on a number of soil characteristics (see ref. ^15^ for further details). Microcosms were planted with two grass species, two legumes species and five forb species representing Swiss grassland communities. Seeds were surface sterilized with 2.5% hyposodium chlorate and germinated on 1% water-agar. The six soil community treatments were replicated eight times, with the exception of the sterile community, which was replicated 10 times for a total of 50 microcosms. Microcosms received a simulated rainfall through purifying filters, which dripped evenly distributed within the microcosm to maintain soil moisture content in the rage of 10–25% (w/w). Microcosms received natural light that was subsidized by 400-W high-pressure sodium lamps to maintain light levels above 300 W/m^2^ for 16 h days at an average of 25 °C and 8 h nights on average of 16 °C.

### Ecosystem functions

We quantified 10 functions known to be linked to the functioning of the soil microbiome that reflect ecosystem nutrient cycling: litter decomposition, soil denitrification through N_2_O emissions, N leaching, P leaching, as well as the N and P uptake by grasses, forbs and legumes. The standing biomass of each plant species was collected after 12 and 24 weeks and pooled by the plant functional group, and the N and P uptake of each of the three plant functional groups (grasses, forbs, and legumes) was assessed. Plant material was ground, and the N content was determined using a CHNSO analyzer (Euro EA, HEKAtech GmbH, Wegberg, Germany). For plant P determination ground biomass was ashed at 600 °C and digested using 6 M HCl. Digests were diluted and P was quantified colorimetrically according to the molybdenum blue method^63^.

Decomposition was quantified using litterbags that were 5 × 5 cm and made of plastic mesh (20 µm mesh). Bags were filled with 1 g of *Lolium multiflorum* shoots that had been sterilized previously. Two litterbags were inserted into each microcosms just below the soil surface. Litter decomposition was measured as the percentage of litter mass that was not recovered from the bags after 24 weeks. Prior to the destructive harvest at 24 weeks, microcosms were watered to 10% above saturation and 50 mL of the leachate was collected and the concentration of P (organic and inorganic P) and N (sum of NH_4_ and NO_3_) within the leachate was determined. N_2_O production was measured at the end of the experiment after fertilizer addition and soil saturation with water. N_2_O fluxes were measured using a TEI 46c automated N_2_O analyzer (Thermo Fisher Scientific, Waltham, USA) for a period of 10 min, three times per day over 3 days starting immediately after the simulated rain prior to harvest at 24 weeks. The N_2_O fluxes were integrated over this period by linear interpolation between single measurements and the total N_2_O emitted was used as an ecosystem function, since N_2_O represents nutrient loss as well as an important greenhouse gas.

### Microbiome profiling

We used next generation sequencing to characterize the soil microbiome. This contrasted to our earlier study^15^, where we used a molecular-profiling method (RISA) that did not allow us to characterize the identity of the microbes. Soil DNA was extracted from 500 mg mixed soil subsamples using the FastDNA^®^ SPIN Kit for Soil (MP Biomedicals, Switzerland) following the manufacturer’s instructions. *PCR*: PCR reactions were conducted with 10 ng of extracted soil DNA as template per 25 µl PCR reaction and the PCR products were run on an electrophoresis gel along with a negative PCR control to verify that the PCR products were not contaminated. Bacterial communities were profiled based on the 16S rRNA gene primers 515F (5′-454ADAPTER-BARCODES[Database S1]-LINKER[GT]-PRIMERSEQ.-3′^64^ and 806R (5′-454ADAPTER-PRIMERSEQ.-3′^65^ with 10x PCR-buffer and MilliQ-purified H_2_O, 5 µm dNTP’s, 1 unit of fast start Exp-Polymerase (Roche), and 5 µm of each forward and reverse primers. PCR cycling conditions were set for 95 °C for 5 min, followed by 25 cycles of denaturation at 95 °C for 30 s, annealing at 53 °C for 1 min, extension at 72 °C for 1 min, with a final extension of 72 °C for 10 min. Fungi communities were quantified with the internal transcribed spacer (ITS) primers fITS9 (5′-454ADAPTER-BARCODES[Database S1]-LINKER[AA]-PRIMERSEQ.-3′^66^) and ITS4 (5′-454ADAPTER-LINKER[CT]-PRIMERSEQ.-3′^67^) with 10x PCR-buffer and MilliQ purified H_2_O, 5 µm dNTP’s, 1 unit of fast start Exp-Polymerase (Roche), and 5 µm of each forward and reverse primers. PCR cycling conditions were set for 95 °C for 5 min, followed by 25 cycles of denaturation at 95 °C for 30 s, annealing at 53 °C for 1 min, extension at 72 °C for 1 min, with a final extension of 72 °C for 10 min. PCR products (pooling five reactions per sample) were purified using QIAquick PCR Purification Kit (Qiagen, Hilden, Germany) following the manufacturers instructions. Purified PCR products were pooled to libraries (1x 16S library, 2x ITS libraries) of equal PCR product amounts and sequenced using 454 sequencing at Microsynth (Balgach, Switzerland).

### Sequence data processing

We employed the QIIME environment (v.1.8.0)^68^ for sequence processing and started the analysis from the raw SFF files (deposited at the European Nucleotide Archive database, accession no. PRJEB22310). The *sffinfo* tool from 454 was used to extract the sequence, quality, and flow files. The script *split_libraries.py* was employed to assign the reads based on their barcodes to their corresponding samples and the reads were filtered for high-quality sequences (minimum length, 200 bp; min. average qual. score, 25; qual_score_window, 50 bp, discarding sequences below the threshold; no ambiguous base calls; no errors in the barcode). The quality sequences were subsequently denoised using the scripts *denoise_wrapper.py* and *inflate_denoiser_output.py*. For delineation of operational taxonomic units (OTUs) we used the UPARSE series of scripts (v8.0.1632_i86linux32)^69^: the denoised quality sequences were trimmed to a common length (16S: 250 bp, ITS: 330 bp), de-replicated, sorted by abundance, singletons excluded, and finally clustered to OTUs of ≥97% sequence similarity. Although the *cluster_otu* function performs a first chimera detection^70^, we additionally screened the OTU representative sequences for chimeric sequences against reference databases (16S^70^, ITS^71^) employing the UCHIME^72^ algorithm. We finally mapped the denoised quality sequences to the chimera-free OTU representative sequences utilizing the *usearch_global* script and default settings. 16S and ITS OTU representative sequences were taxonomically assigned employing the RDP classifier^71^ against the SILVA (release 119, 97% OTUs)^72^ and the UNITE (v7, 01.08.2015, dynamic OTUs)^73^ databases, respectively. We removed OTUs that were not classified as bacteria or fungi or unclassified at domain/kingdom level from the OTU tables. For all following calculations, the OTUs tables were converted to proportions of total reads per sample and the OTUs that comprised <1% of the total number of reads on average were disregarded. Of these, OTUs that were not present within at five or fewer experimental replicates were also omitted from the data set. The OTU matrices were then standardized (each OTU has an overall mean of 0 and unit variance).

### Microbial networks

Microbial association networks for each microcosm were created by first creating a network meta-matrix using both the standardized fungal and bacterial OTU tables (resulting in an 1814^2^ OTUs sparse matrix). The meta-matrix was generated using the R package “SpiecEasi”, which uses LASSO regularization and cross-validation to detect the most parsimonious network structure in high dimensional microbial data^35^. The lambda ratio was 0.01 and the network was assessed over 50 values of lambda for each 100 cross-validation permutations to detect the least variable network links by StARS selection criterion^73^. The networks were estimated at each permutation by the Meinshausen and Bühlmann graph estimation method^74^. The network meta-matrix was then used to sub-set networks matrices for each microcosm by selecting out OTUs that were detected to be present within the microcosm. From these sub-networks, the number of links was counted, and complexity was calculated as linkage density (links per OTU)^75^ among bacteria, fungi, fungi-bacterial only, or all fungal and bacteria OTUs. We also calculated the network functional complexity (same as before but using only taxa that are not only associated with one another, but that also have positive, or negative, coefficients for predicting the same function to define an index of positive network functional complexity.

### Taxonomic compositions that predict functions

We used randomization tests to assess whether microbial taxa can predict particular ecosystem functions based on effect sizes and the standard 95% confidence range following previous studies^76,77^. However, this method considers taxa independently of one another. Therefore, we also used elastic net regularization, which allowed us to consider all taxa simultaneously as predictors within a large sparse dataset, where the number of predictors is much greater than the number of observations^33^. Instead of assigning significance values to each taxa separately using randomization tests, this ‘feature selection’ method selects taxa (predictors) that achieve the best prediction of an ecosystem function by penalizing the coefficient of each taxa by mixing *λ*_1_ (LASSO regression) and *λ*_2_ (ridge regression) type penalties along a gradient of *α* (where *α* *=* 0 uses solely a type *λ*_1_ penalty and *α* *=* 1 uses solely a type *λ*_2_ penalty)^33^. Therefore, the method selects out fungal and bacterial OTU combinations that together best predict the response in ecosystem functioning. This was done using the R package ‘glmnet’^33^. We assessed models across a gradient of *α* ranging from 0 to 1 in steps of 0.01 using the function ‘cv.glmnet’ with leave-one-out cross validation (*k*-folds = *n*). The *α* and corresponding *λ* values that resulted in the minimum MSE were used in the final model to obtain coefficients for each OTU. The non-zero coefficients were then used to infer a positive or negative contribution of an OTU to improving ecosystem functioning in the desired direction. Both randomization tests and elastic net methods produced similar results (see Table 1, Supplementary Fig. 1 and Supplementary Table 6), and here we focus on the results using elastic net regularization where all taxa were considered simultaneously.

### Analyses

All community characteristics and all ecosystem functions were assessed for variation among treatments by ANOVA. We further tried to discount for the effect of the most extreme ‘Sterile’ soil treatment by including it as a contrast (sterile vs. other treatments) term within the AMOVA model. However, discounting for the ‘Sterile’ soil treatment first generally had little effect on our results (see Supplementary Tables 1 and 2). Each function was regressed on each of the microbiome community characteristics independently. To summarize the changes in the overall functioning of soils in relation to soil community characteristics, we calculated a multifunctionality index by averaging the normalized (zero mean and unit variance) ecosystem function measures (forb N and P uptake, legume N and P uptake, grass N and P uptake, decompositions, N and P leaching and N_2_O emissions). However, since many functions are correlated, we also calculated the multidimensional multifunctionality index recently described to avoid potential co-linearity issues^78^. Although there are many methods by which to assess multifunctionality and its relation to biodiversity, these methods conveniently provide a single index that reflects the general functioning of the ecosystem on average^78,79^. Here both the averaging and multidimensional indices of multifunctionality were highly correlated (Pearson’s rho = 0.882, *P* < 0.001). Thus, we only present results using the multidimensional calculation of multifunctionality. Prior to calculating multifunctionality, values for nutrient leaching and N_2_O emissions were inverted as higher values of these functions are undesired ecosystem functions (i.e. low values for nutrient leaching indicate the retention of nutrients and proper ecosystem functioning, while high values indicate dysfunction and nutrient losses).

### Reporting summary

Further information on research design is available in the Nature Research Reporting Summary linked to this article.

## Supplementary information

Supplementary Information

Reporting Summary

## Data Availability

Data are publicly available at 10.6084/m9.figshare.9767423.v1. The data used for this study are available in ‘figshare’ with the identifier https://doi.org/10.6084/m9.figshare.9767423.v1.

## Code availability

R code is available upon request.
