# Supplementary material for: Fungal-bacterial diversity and microbiome complexity predict ecosystem functioning
Source: Nat Commun. 2019 Oct 24;10:4841. doi: 10.1038/s41467-019-12798-y (PMC6813331; doi:10.1038/s41467-019-12798-y)
Supplement: Supplementary file 1 — Supplementary Information [file 41467_2019_12798_MOESM1_ESM.pdf]

Supplementary Materials

Fungal-bacterial diversity and microbiome complexity predict ecosystem functioning

Cameron Wagg, Klaus Schlaeppi, Samiran Banerjee, Eiko E. Kuramae, Marcel G. A. van der Heijden

This file contains:

- Figure S1. Taxonomy and richness of soil bacteria and fungi.
- Figure S2. Relationships between richness and individual ecosystem functions
- Figure S3. Number of taxa associated with number of functions.
- Figure S4. Relationships between number of taxa supporting each function and the functional response
- Figure S5. Relationships of fungal and bacterial network functional complexity with individual ecosystem functions
- Figure S6. Relationships of fungal-bacterial network functional complexity with individual ecosystem functions
- Table S1. Microbial community characteristics for each soil treatments
- Table S2. Microbial community characteristics per soil treatment after de-trending for richness.
- Table S3. ANOVA results for effects of richness on individual functions and the residual unexplained variation among treatments.
- Table S4. Taxonomy of soil fungi and bacteria linked to functions.
- Table S5. Similarity among functions in the microbial taxa they are linked to.
- Table S6. Effects of soil microbial community characteristics on multifunctionality using randomization tests.

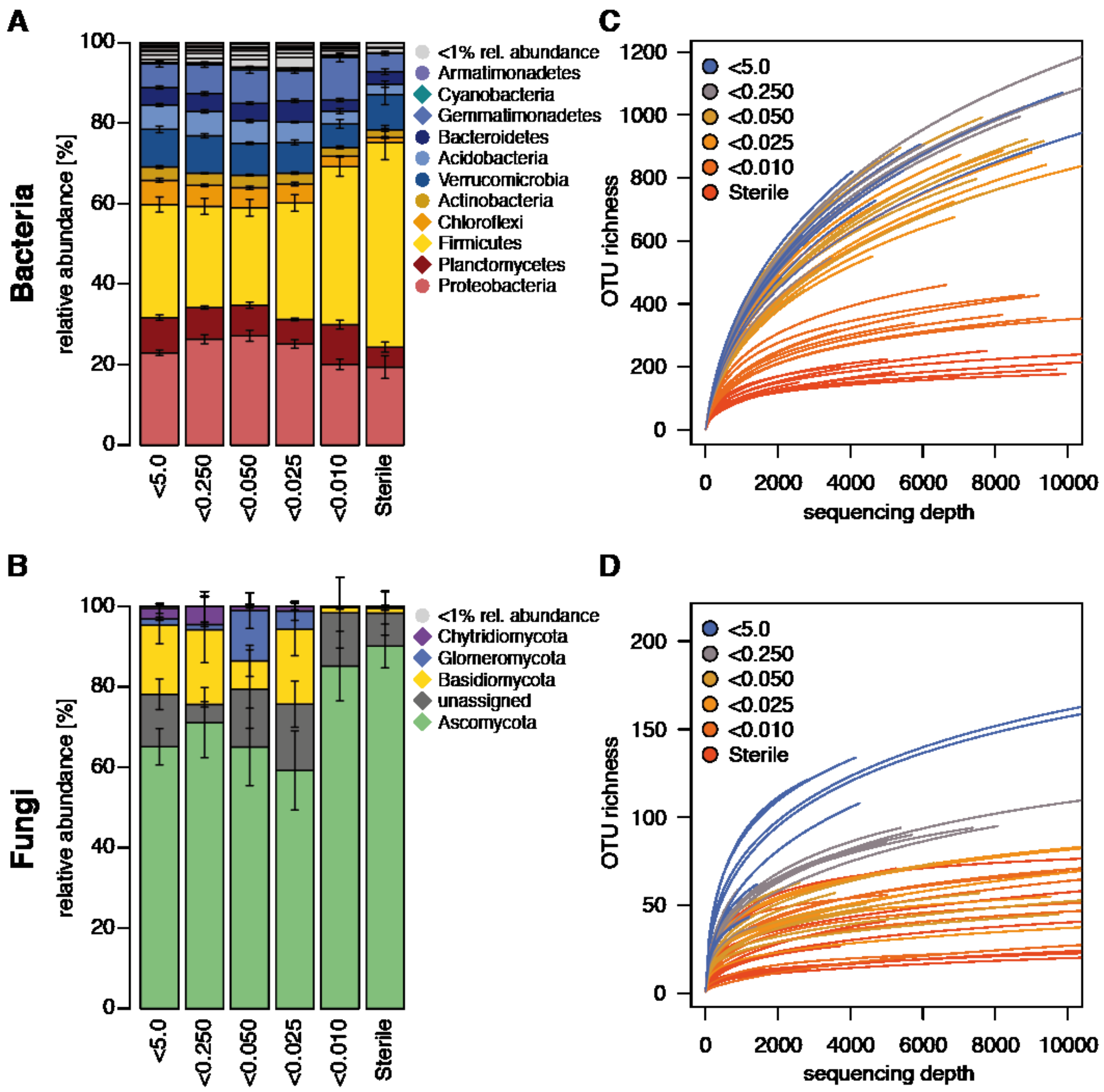

Fig. S1. Taxonomy and richness of soil bacteria and fungi. The taxonomic composition of bacteria (per class) (**A**, **C**) and fungi (per phylum) (**B, D**) in microcosms with progressive filtering of soil biota. (**A**, **B**). Phyla with mean (±s.e.m.) relative abundances >1% are shown and Phyla varying significantly between treatments are indicated with a diamond symbol in the colour legend (ANOVA-like likelihood ratio test, $P<0.05$ (FDR)). (**C**, **D**) Rarefaction curves depicting OTU richness as a function of sequencing depth were constructed by sub-sampling in increasing steps of 100 sequences per sample (the graphs were cropped at 10,000 sequences per sample for clarity). Individual samples are colored by treatment groups.

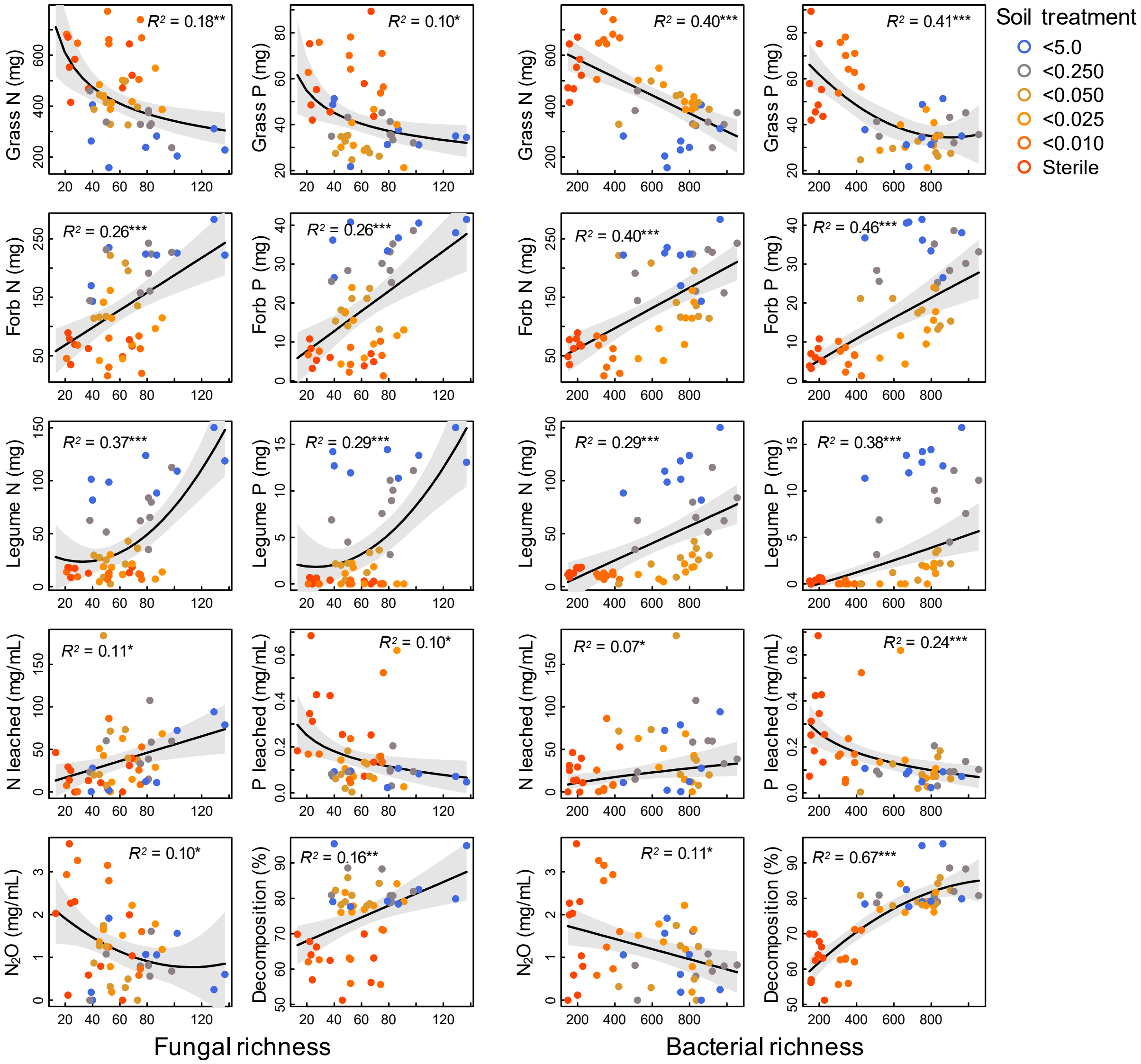

Fig. S2. Relationships between microbial richness and ecosystem functions. Relationships between fungal and bacterial OTU richness and ecosystem functions are shown. Points are colored by the filtering treatment. Solid lines are regression fits with the shaded region indicating the 95% confidence band. Relationships were fit using the best fitting regression (based on adjusted R^2 shown within each panel) using linear, second order polynomial and power function (log-log). Significance for the fit is indicated by $*P < 0.05$, $**P < 0.01$ and $***P < 0.001$.

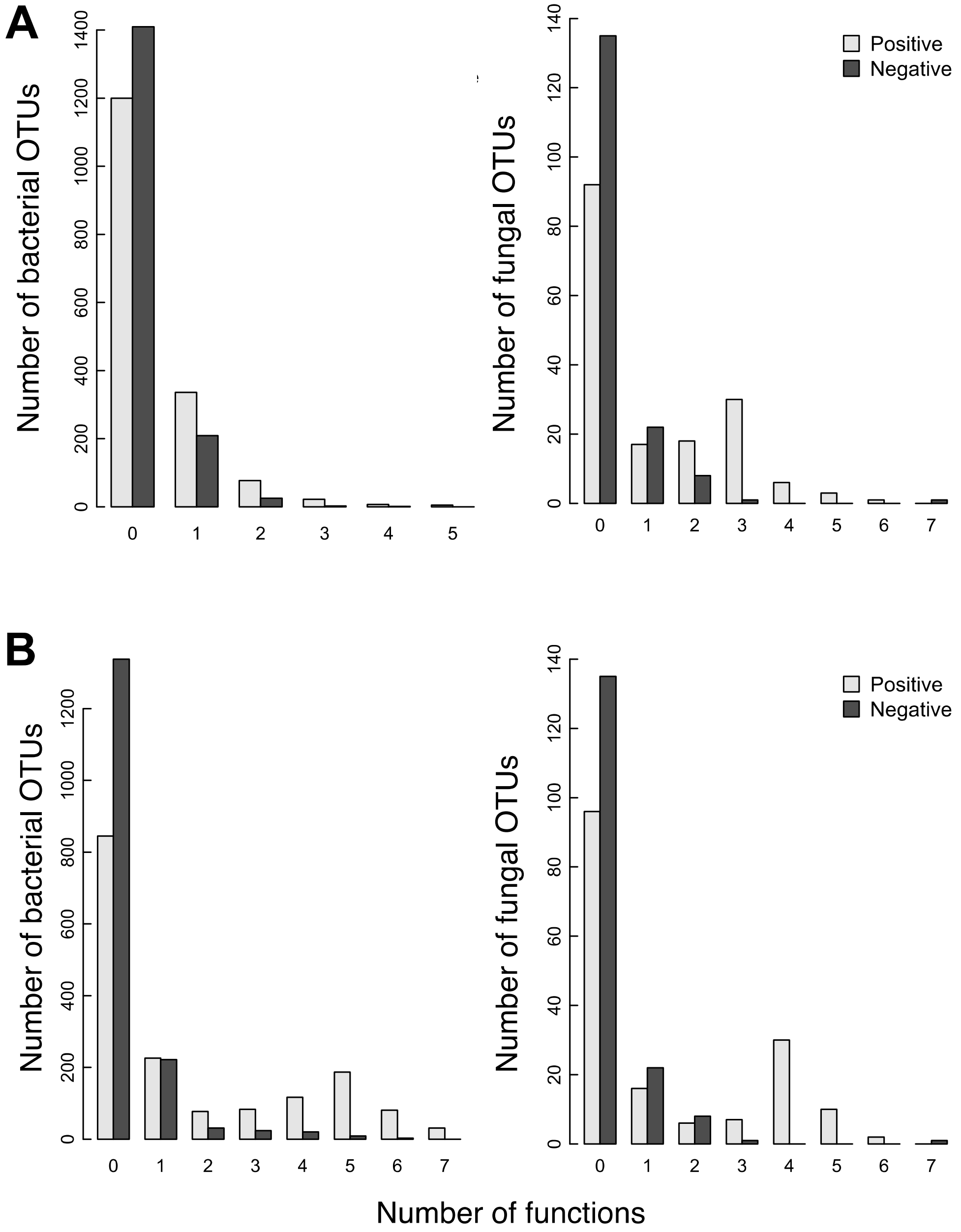

Fig. S3. Number of taxa associated with number of functions. (A) Using the elastic net method to simultaneously identify taxa that together best predict ecosystem functions and (B) using randomization tests considering each taxa independently to identify taxa that significantly (95% CI) relate to a function.

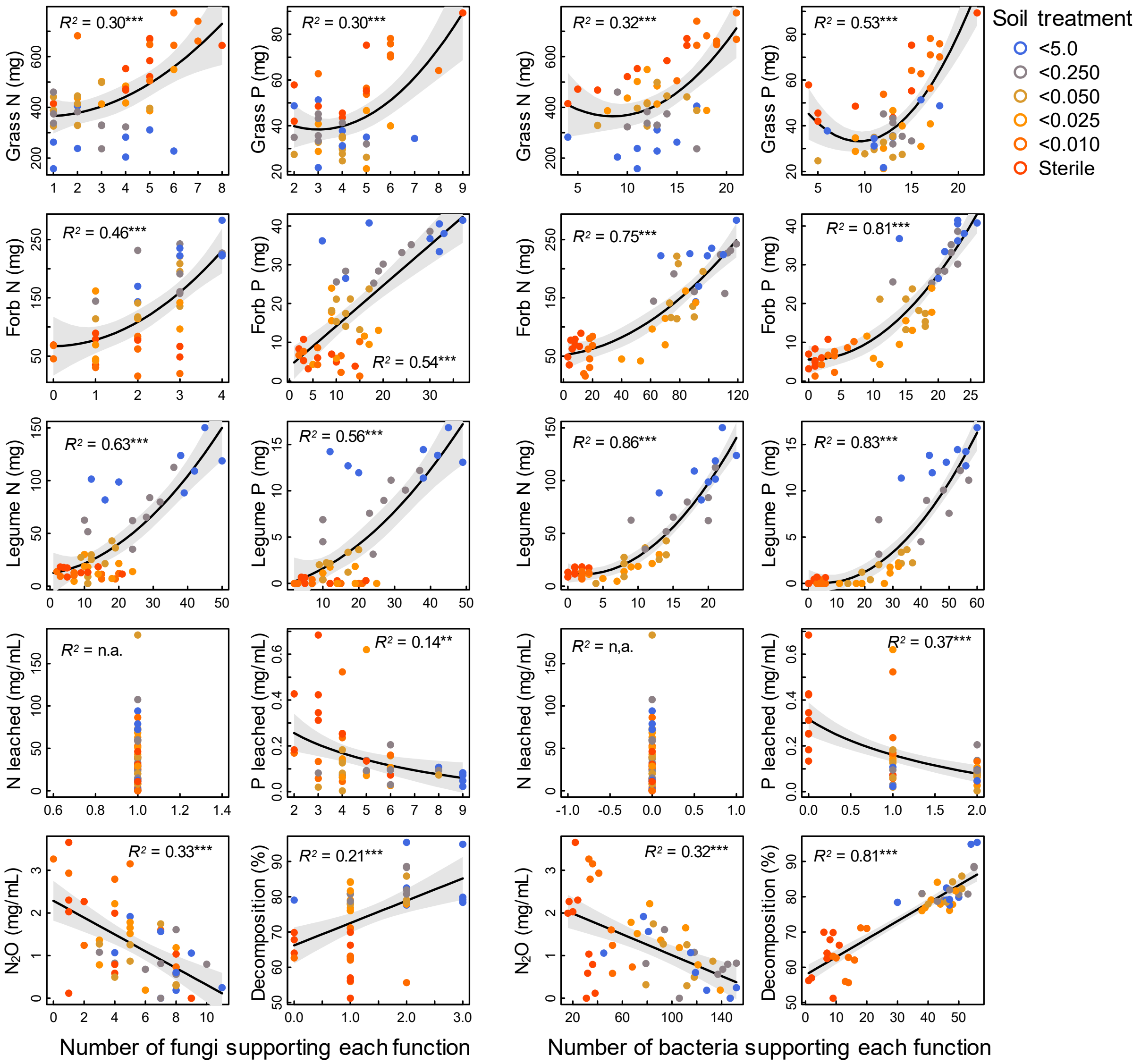

Fig. S4. Relationships between each ecosystem function and the number of taxa present found to support each function. Relationships between the number of fungal and bacterial OTUs present that were found to support each function and the ecosystem function itself are shown. Points are colored by the filtering treatment. Solid lines are regression fits with the shaded region indicating the 95% confidence band. Relationships were fit using the best fitting regression (based on adjusted R^2 shown within each panel) using linear, second order polynomial and power function (log-log). Significance for the fit is indicated by $*P < 0.05$, $**P < 0.01$ and $***P < 0.001$. Note that there was only one fungal OTU detected who's abundance could predict a reduction in N leaching, which was an OTU present in every microcosm. Resultantly there are no relationships between N leaching and the taxa that best predict its reduction as indicated by R^2 = n.a.

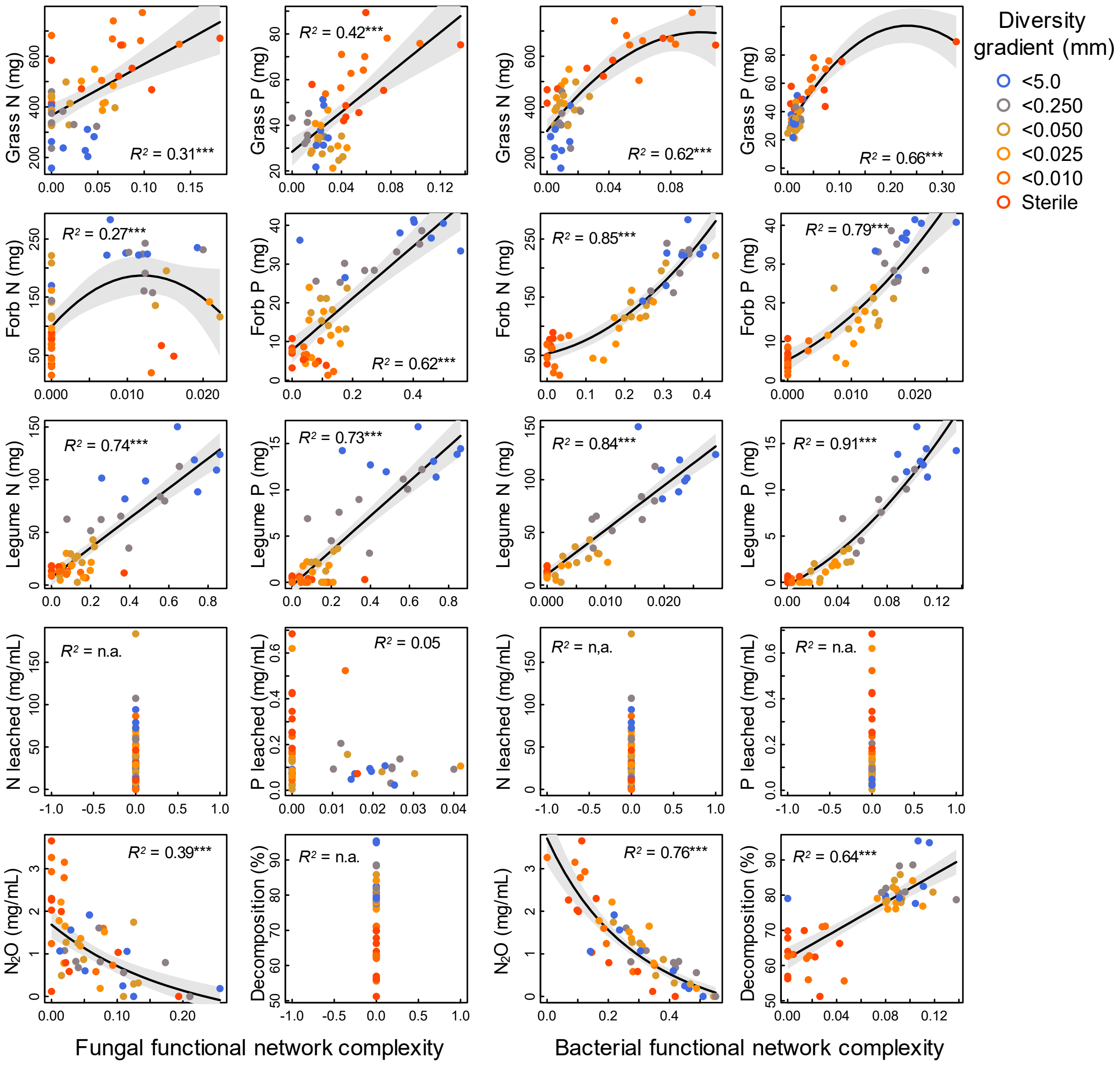

Fig. S5. Relationships between each ecosystem function and the fungal-fungal and bacterial-bacterial network complexity among taxa that support each function. Points are colored by the filtering treatment. Solid lines are regression fits with the shaded region indicating the 95% confidence band. Relationships were fit using the best fitting regression (based on adjusted R^2 shown within each panel) using linear, second order polynomial and power function (log-log). Significance for the fit is indicated by $*P < 0.05$, $**P < 0.01$ and $***P < 0.001$.

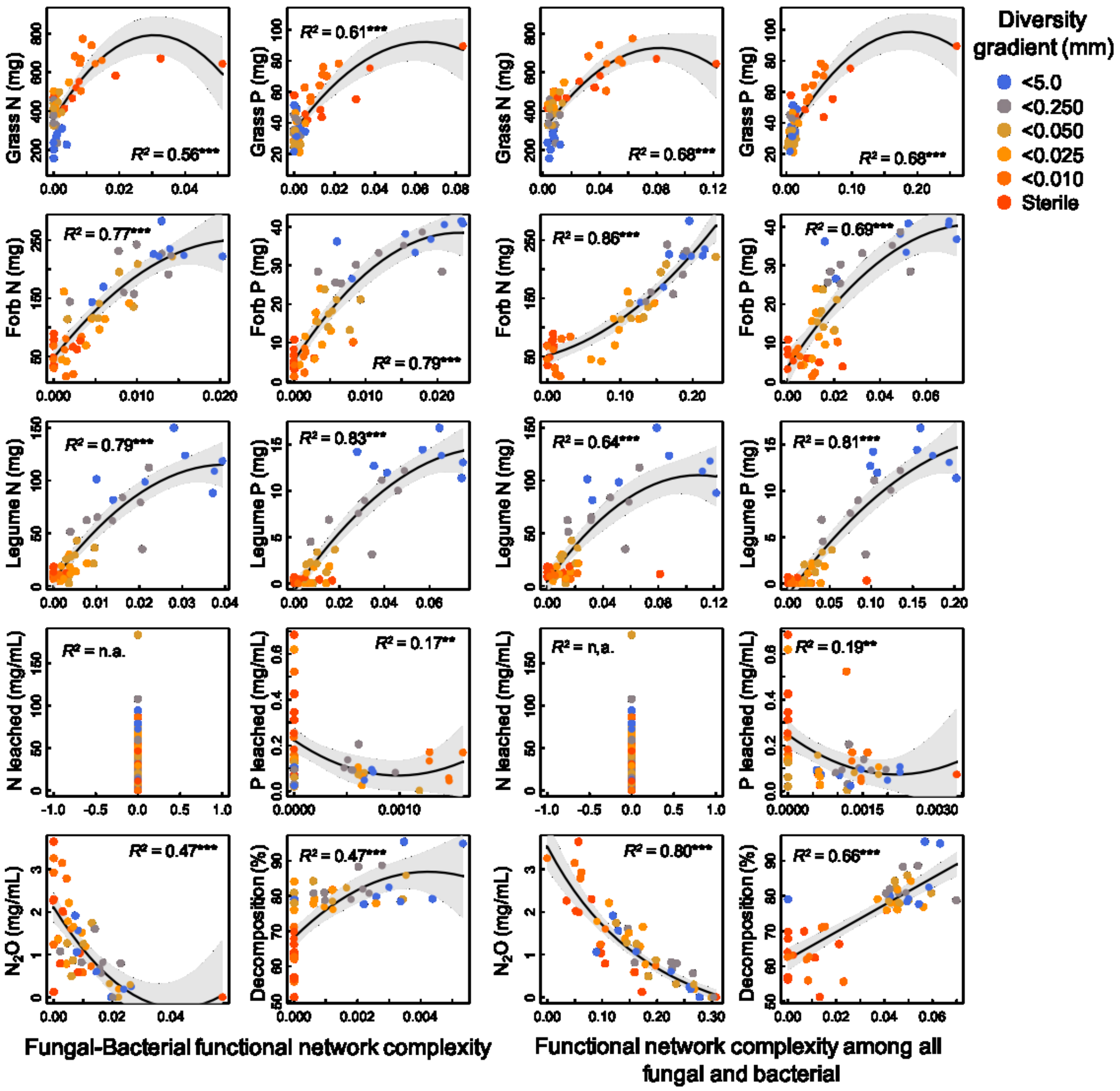

Fig. S6. Relationships between each ecosystem function and the network complexity among fungi-bacteria only and among all fungi and bacteria taxa that support each function. Points are colored by the filtering treatment. Solid lines are regression fits with the shaded region indicating the 95% confidence band. Relationships were fit using the best fitting regression (based on adjusted R^2 shown within each panel) using linear, second order polynomial and power function (log-log). Significance for the fit is indicated by *$P < 0.05$, **$P < 0.01$ and ***$P < 0.001$.

Table S1. Microbial community characteristics for each soil treatments. The mean of each sieving treatment is provided. The F-ratio and degrees of freedom are provided for the variation across all treatments. § is the F-ratio and degrees of freedom for the variation among treatments after first accounting for the 'Sterile' treatment. Significance is indicated by * $P < 0.05$, ** $P < 0.01$, *** $P < 0.001$.

	$F_{5, 37}$	§$F_{4, 37}$	< 5.0	< 0.250	< 0.050	< 0.025	< 0.010	Sterile
**Bacteria**								
Richness	***44.8	***20.2	740.25	817.63	738.63	728.13	357.25	183.73
Positive	***52.3	***28.9	248.63	261.88	226.25	202.38	91.13	49.87
Negative	***22.8	2.6	108.38	116.38	116.63	126.75	104.25	58.69
Uniqueness	***23.7	***20.8	0.097	0.088	0.075	0.065	0.043	0.046
Links	***28.0	***15.2	7347.88	9027.00	7463.38	7174.50	2419.50	880.03
Complexity	***46.9	***18.5	9.67	10.67	9.84	9.74	6.73	4.82
+ Complexity	***40.3	***31.7	0.94	0.95	0.81	0.65	0.33	0.33
- Complexity	**12.8	***14.5	0.25	0.23	0.26	0.31	0.67	0.46
**Fungi**								
Richness	**3.9	2.4	83.13	73.50	55.00	63.50	53.75	39.70
Positive	***5.9	**5.3	44.25	34.38	23.75	25.50	22.50	18.44
Negative	1.8	1.6	15.63	12.38	9.88	13.88	13.75	10.23
Uniqueness	**4.8	***6.0	0.261	0.213	0.177	0.115	0.152	0.192
Links	**5.1	**4.0	203.00	160.88	101.88	119.00	94.75	65.31
Complexity	***6.8	**4.2	2.34	2.14	1.82	1.84	1.65	1.39
+ Complexity	***18.3	***19.3	1.76	1.18	0.54	0.44	0.42	0.36
- Complexity	***8.5	**4.2	0.10	0.07	0.08	0.12	0.18	0.22
**Fungi-Bact.**								
Richness†	***21.9	***9.61	0.65	0.66	0.55	0.58	0.37	0.23
Positive†	***35.3	***23.0	0.67	0.60	0.45	0.42	0.22	0.20
Negative†	***8.2	1.2	0.69	0.65	0.63	0.73	0.67	0.46
Uniqueness	***8.1	***10.0	0.178	0.135	0.108	0.082	0.099	0.111
Links	***13.5	***8.0	656.69	652.00	457.19	509.50	222.06	117.50
Complexity	*3.5	*2.8	0.78	0.72	0.58	0.65	0.53	0.50
+ Complexity	***17.8	***21.2	0.23	0.15	0.08	0.06	0.06	0.08
- Complexity	***7.6	*3.4	0.03	0.03	0.03	0.04	0.08	0.10
**Network characteristic among all taxa**								
Links	***28.4	***15.4	8207.56	9839.88	8022.44	7803.00	2736.31	1062.84
Complexity	***48.3	***19.7	9.73	10.68	9.86	9.75	6.63	4.77
+ Complexity	***44.4	***39.9	1.28	1.13	0.87	0.69	0.40	0.43
- Complexity	***11.7	***12.5	0.27	0.24	0.27	0.33	0.69	0.52

Note: All richness measures are number of OTUs ('Richness', Richness of OTUs with 'Positive' or 'Negative' coefficients to ecosystem functions). Links are number of links between OTUs (among 'Bacteria' only, 'Fungi' only, 'Fungi-Bacteria' only, or among 'All' fungi and bacteria). Complexity refers to the number of network links per OTUs (nodes). † indices are the average of the scaled (between 1 and 0) values of fungi and bacteria.

Table S2. Microbial network complexity characteristics for each soil treatments after de-trending for richness. The mean of each sieving treatment is provided. The *F*-ratio and degrees of freedom are provided. *§* is the *F*-ratio and degrees of freedom for the variation among treatments after first accounting for the ‘Sterile’ treatment. Significance is indicated by * $P < 0.05$, ** $P < 0.01$, *** $P < 0.001$.

	$F_{5, 37}$	§$F_{4, 37}$	< 5.0	< 0.250	< 0.050	< 0.025	< 0.010	Sterile
**Bacteria**								
Complexity	***5.8	***6.2	8.24	8.55	8.42	8.41	8.69	8.31
+ Complexity	***5.7	***7.2	0.81	0.75	0.68	0.52	0.52	0.66
- Complexity	**4.8	**5.3	0.32	0.33	0.32	0.37	0.58	0.30
**Fungi**								
Complexity	1.7	1.2	1.99	1.94	1.90	1.79	1.75	1.70
+ Complexity	***11.8	***14.7	1.36	0.96	0.64	0.39	0.54	0.71
- Complexity	**4.9	*3.0	0.13	0.08	0.08	0.12	0.17	0.20
**Fungi-Bact.**								
Complexity	2.3	2.0	0.62	0.63	0.62	0.62	0.58	0.64
+ Complexity	***15.5	***18.7	0.19	0.13	0.09	0.05	0.07	0.13
- Complexity	0.5	0.3	0.05	0.05	0.04	0.05	0.06	0.06
**All**								
Complexity	**3.9	**3.9	8.28	8.53	8.41	8.40	8.62	8.32
+ Complexity	***10.2	***12.6	1.04	0.89	0.80	0.57	0.59	0.83
- Complexity	**3.8	*4.3	0.37	0.35	0.32	0.39	0.60	0.34

Note: All richness measures are number of OTUs (‘Richness’, Richness of OTUs with ‘Positive’ or ‘Negative’ coefficients). All network characteristics are number of links between OTUs (among ‘Bacteria’ only, ‘Fungi’ only, ‘Fungi-Bacteria’ only, or among ‘All’ fungi and bacteria). Complexity refers to the number of network links per number of OTUs (nodes). † indices are the average of the scaled (between 1 and 0) values of fungi and bacteria.

Table S3. ANOVA results for each ecosystem function and their composite multifunctional variable in relationship to fungal and bacterial OTU richness. Relationships were fit using linear regression (Linear) second order polynomial (Poly) and power function (Power). The sieving treatment was fit after to capture the residual variation among treatments in functioning that is not explained by fungal and bacterial richness and is highlighted in bold when significant ($P < 0.05$).

	Bacterial richness					Fungal richness				
Function	Terms	*Df*	*MS*	*F*	*P*	Terms	*Df*	*MS*	*F*	*P*
Grass N	Linear	1	428084.70	82.29	<0.001	Power	1	1.19	36.71	<0.001
	**Treat.**	**5**	**79304.32**	**15.24**	**<0.001**	**Treat.**	**5**	**0.68**	**21.03**	**<0.001**
	Resid.	41	5202.13			Resid.	41	0.03		
Grass P	Poly	2	2711.44	28.13	<0.001	Power	1	0.68	16.41	<0.001
	**Treat.**	**5**	**651.02**	**6.75**	**<0.001**	**Treat.**	**5**	**0.63**	**15.24**	**<0.001**
	Resid.	40	96.41			Resid.	41	0.04		
Forb N	Linear	1	102485.80	73.10	<0.001	Linear	1	68987.72	54.76	<0.001
	**Treat.**	**5**	**17918.36**	**12.78**	**<0.001**	**Treat.**	**5**	**25784.53**	**20.47**	**<0.001**
	Resid.	41	1402.02			Resid.	41	1259.76		
Forb P	Power	1	13.34	101.90	<0.001	Linear	1	2045.12	107.06	<0.001
	**Treat.**	**5**	**1.94**	**14.83**	**<0.001**	**Treat.**	**5**	**913.95**	**47.85**	**<0.001**
	Resid.	41	0.13			Resid.	41	19.10		
Legume N	Linear	1	22193.08	154.00	<0.001	Poly	2	14468.44	86.80	<0.001
	**Treat.**	**5**	**8903.54**	**61.78**	**<0.001**	**Treat.**	**5**	**7402.93**	**44.41**	**<0.001**
	Resid.	41	144.11			Resid.	40	166.69		
Legume P	Power	1	20.01	232.19	<0.001	Poly	2	211.76	84.97	<0.001
	**Treat.**	**5**	**5.59**	**64.91**	**<0.001**	**Treat.**	**5**	**158.62**	**63.64**	**<0.001**
	Resid.	41	0.09			Resid.	40	2.49		
N leaching	Power	1	7.21	4.50	0.040	Linear	1	7914.27	7.44	0.009
	Treat.	5	1.22	0.76	0.582	Treat.	5	1295.12	1.22	0.317
	Resid.	43	1.60			Resid.	43	1063.82		
P leaching	Power	1	0.17	16.96	<0.001	Power	1	0.08	7.68	0.008
	Treat.	5	0.01	1.23	0.314	**Treat.**	**5**	**0.03**	**3.02**	**0.020**
	Resid.	43	0.01			Resid.	43	0.01		
N_2O	Linear	1	5.24	7.31	0.010	Poly	2	2.70	4.02	0.025
	Treat.	5	1.03	1.43	0.232	Treat.	5	1.52	2.26	0.066
	Resid.	43	0.72			Resid.	42	0.67		
Decomposition	Poly	2	1771.26	66.48	<0.001	Linear	1	932.97	33.60	<0.001
	**Treat.**	**5**	**108.34**	**4.07**	**0.004**	**Treat.**	**5**	**615.23**	**22.15**	**<0.001**
	Resid.	42	26.64			Resid.	43	27.77		
Multifunction.	Linear	1	2.04	288.53	<0.001	Linear	1	1.28	193.5	<0.001
	**Treat.**	**5**	**0.33**	**46.09**	**<0.001**	**Treat.**	**5**	**0.48**	**72.3**	**<0.001**
	Resid.	41	0.01			Resid.	41	0.01		

Table S4. Taxonomy of soil fungi and bacteria linked to functions. Number of fungal OTUs per fungal class with positive/negative coefficients for predicting a given ecosystem function (by column). Note that positive here refers to reducing nutrient leaching and N_2O emissions.

**Fungal class**	**N**	**Decom.**	**N_2O**	**P leach.**	**N leach.**	**Leg. N**	**Gr. N**	**Forb N**	**Leg. P**	**Gr. P**	**Forb P**
Agaricomycetes	55	1/1	5/1	0/3	0/1	13/0	2/0	0/0	12/0	3/0	9/0
Chytridiomycetes	7	0/0	1/0	0/0	0/1	3/0	0/1	1/0	3/0	0/2	3/0
Cystobasidiomycetes	1	0/0	0/0	0/0	0/0	1/0	0/1	0/0	1/0	0/0	1/0
Dothideomycetes	14	0/1	2/2	1/4	1/0	3/0	2/0	1/0	3/0	4/0	3/2
Eurotiomycetes	13	0/0	0/1	1/3	0/0	4/0	2/1	1/0	4/0	0/0	3/0
Glomeromycetes	5	1/0	1/0	1/0	0/0	2/0	0/1	0/0	2/0	0/0	1/0
Lecanoromycetes	3	0/0	0/0	1/1	0/0	1/0	0/0	0/0	1/0	0/0	0/0
Leotiomycetes	4	0/0	0/0	0/0	0/0	1/0	0/0	0/0	1/0	0/0	1/0
Orbiliomycetes	3	0/0	0/0	0/1	0/0	1/0	0/1	0/0	1/0	0/0	1/0
Pezizomycetes	11	0/0	1/0	1/0	0/1	4//0	0/1	0/0	5/0	0/0	2/0
Pucciniomycetes	3	0/0	0/0	0/0	0/0	1/0	0/0	0/0	1/0	0/0	1/0
Saccharomycetes	7	0/0	0/0	0/0	0/2	2/0	1/0	0/0	2/0	1/0	1/0
Sordariomycetes	37	1/1	2/4	4/3	0/0	13/0	1/1	1/0	13/0	1/0	9/2
Tremellomycetes	4	0/0	0/0	0/0	0/0	3/0	0/1	0/0	3/0	0/0	3/0

**Bacterial class**	**N**	**Decom.**	**N_2O**	**P leach.**	**N leach.**	**Leg. N**	**Gr. N**	**Forb N**	**Leg. P**	**Gr. P**	**Forb P**
Actinobacteria	128	4/1	15/12	0/0	0/0	3/0	1/0	15/3	10/0	0/2	6/2
Alphaproteobacteria	167	7/4	24/15	1/2	0/1	1/2	4/4	14/4	5/4	4/1	2/2
Anaerolineae	9	0/0	1/0	0/0	0/0	0/0	0/1	0/0	0/0	0/0	0/0
Aquificae	4	1/0	0/0	0/0	0/0	1/0	0/1	1/0	1/0	0/0	0/0
Armatimonadia	1	0/0	0/0	0/1	0/0	0/0	0/0	0/0	0/0	0/0	0/0
Bacilli	131	4/5	15/19	0/1	0/0	1/0	3/0	10/1	4/0	3/0	3/0
Bacteroidia	13	0/0	5/3	0/0	0/0	0/0	0/0	2/0	1/0	0/0	0/0
Betaproteobacteria	63	1/0	6/12	0/0	0/0	3/0	1/2	5/1	5/0	1/1	1/0
Caldilineae	9	0/0	2/1	0/0	0/0	0/0	0/0	1/0	1/0	0/0	0/0
Chlamydiae	20	0/0	2/0	0/0	0/0	0/1	0/0	0/0	0/1	0/0	0/0
Chlorobia	1	0/0	0/0	0/0	0/0	0/0	0/0	0/0	1/0	0/0	0/0
Chloroflexi	5	0/0	0/1	0/0	0/0	0/0	0/0	0/0	0/0	0/0	0/0
Clostridia	208	6/0	31/15	1/0	0/3	2/0	3/3	18/6	5/2	4/4	2/1
Deferribacteres	1	0/0	0/0	0/0	0/0	0/0	0/0	0/0	0/0	0/0	0/0
Deltaproteobacteria	174	4/2	33/11	0/0	0/1	4/0	3/0	13/1	10/1	2/2	3/0
Epsilonproteobacteria	4	0/0	0/0	0/0	0/1	0/0	0/0	0/0	0/0	0/0	0/0
Erysipelotrichia	14	1/0	0/1	0/0	0/0	0/0	0/0	1/1	0/0	0/0	0/0
Fibrobacteria	4	0/0	0/1	0/0	0/0	0/0	0/0	0/0	0/0	0/0	0/0
Flavobacteria	4	0/0	0/0	0/0	0/0	0/0	0/0	0/0	0/0	0/0	0/0
Gammaproteobacteria	167	7/1	23/18	0/1	0/1	4/0	0/3	15/1	9/0	2/2	3/0
Gemmatimonadetes	41	3/0	8/7	0/0	0/0	2/0	0/0	8/0	3/0	1/1	1/0
Holophagae	1	0/0	1/0	0/0	0/0	0/0	0/0	0/0	0/0	0/0	0/0
Ignavibacteria	2	0/0	0/0	0/0	0/0	0/0	0/0	0/0	0/0	0/0	0/0
Ktedonobacteria	2	0/0	0/0	0/0	0/0	0/0	0/0	0/0	1/0	0/0	0/0
Mollicutes	11	0/0	2/0	0/0	0/0	0/0	0/0	1/0	0/0	0/0	0/0
Negativicutes	20	1/0	3/0	0/0	0/0	0/0	0/0	0/0	2/0	0/0	0/0

Nitrospira	8	0/0	3/0	0/0	0/0	0/0	0/0	2/0	0/1	0/0	0/0
Opitutae	32	1/1	5/4	0/0	0/0	1/0	2/2	4/2	1/1	1/0	3/0
Phycisphaerae	13	2/0	1/0	0/0	0/0	1/0	0/0	2/0	3/0	0/0	0/0
Planctomycetacia	226	9/3	27/22	0/0	0/1	1/0	6/2	10/4	4/2	5/1	0/1
Sphingobacteria	74	2/1	10/2	0/0	0/1	1/0	3/0	3/2	3/0	2/0	1/0
Spirochaetes	12	0/0	0/0	0/0	0/0	0/0	1/0	1/0	2/0	1/0	0/0
Synergistia	11	0/0	1/1	0/0	0/0	0/0	0/0	1/0	0/0	0/0	0/0
Thermodesulfobacteria	1	0/0	0/1	0/0	0/0	0/0	0/0	0/0	0/0	0/0	0/0
Thermomicrobia	12	1/1	0/0	0/0	0/0	0/0	0/0	0/2	0/0	1/0	0/0
Verrucomicrobiae	54	4/0	12/6	0/0	0/0	1/0	1/0	9/0	4/0	0/1	2/1

Table S5. Similarity among functions in the microbial taxa they are linked to. Shown are the ten different ecosystem functions (diagonal) assessed in this study. Values represent percent (%) overlap among microbial taxa (OTUs) in affecting two different ecosystem functions for combinations of taxa that have positive coefficients (upper triangle) or negative coefficients (lower triangle) in predicting each ecosystem function. For instance, litter decomposition (Decom.) is associated with 7.64 % of the same microbial taxa that are associated with reducing N_2O emissions.

Positive

**Decom.**	7.64	3.33	0.00	14.29	0.00	18.56	15.04	0.00	14.12
4.68	**N_2O**	0.86	0.00	2.34	3.10	15.30	7.87	2.33	3.11
0.00	2.55	**P leach**	0.00	0.00	0.00	1.45	0.00	0.00	0.00
0.00	0.00	0.00	**N leach**	0.00	0.00	0.00	0.00	0.00	0.00
0.00	0.00	0.00	0.00	**Leg N**	0.00	18.52	51.49	0.00	30.19
0.00	0.00	0.00	0.00	0.00	**Gr N**	0.00	0.00	54.55	0.00
0.00	10.00	0.00	0.00	6.45	0.00	**Forb N**	28.44	0.00	25.77
0.00	2.41	0.00	8.70	23.53	0.00	4.76	**Leg P**	0.00	23.53
0.00	0.00	0.00	8.33	0.00	30.30	0.00	0.00	**Gr P**	0.00
0.00	2.53	0.00	0.00	22.22	0.00	29.41	10.00	0.00	**Forb P**

Negative

Table S6. Effects of soil microbial community characteristics on multifunctionality using randomization tests. Based on associating OTUs to functions using randomizations test considering each taxa independently (instead of elastic net considering all taxa simultaneously). Effects are shown before and after de-trending community characteristics for richness prior to analysis. Standardized effect (*SES*) and associated standard error (*SE*), R^2 and *AICc* for each community characteristic as an independent predictor. † average z-transformed values of bacteria and fungi.

	SES	*SE*	*P*	R^2	*AICc*
**Functional richness**					
Bacteria (+)	0.842	0.083	< 0.001	0.693	85.1
Fungi (+)	0.757	0.097	< 0.001	0.568	101.4
†Both (+)	0.902	0.068	< 0.001	0.792	66.4
Bacteria (-)	0.460	0.136	0.002	0.199	131.1
Fungi (-)	-0.105	0.150	0.489	0.010	141.2
†Both (-)	0.245	0.151	0.111	0.054	139.1
**Functional richness (post de-trending)**					
Bacteria (+)	0.565	0.120	< 0.001	0.327	122.8
Fungi (+)	0.599	0.115	< 0.001	0.370	119.6
†Both (+)	0.538	0.124	< 0.001	0.291	125.3
Bacteria (-)	-0.418	0.133	0.003	0.176	132.4
Fungi (-)	-0.620	0.117	< 0.001	0.379	118.9
†Both (-)	-0.443	0.134	0.002	0.193	131.5
**Functional complexity**					
Bact-Bact (+)	0.921	0.059	< 0.001	0.840	53.9
Fungi-Fungi (+)	0.772	0.093	< 0.001	0.601	97.6
Bact-Fungi (+)	0.833	0.079	< 0.001	0.708	82.7
Among all (+)	0.927	0.057	< 0.001	0.852	50.2
Bact-Bact (-)	-0.778	0.091	< 0.001	0.613	96.2
Fungi-Fungi (-)	-0.657	0.113	< 0.001	0.423	115.3
Bact-Fungi (-)	-0.676	0.108	< 0.001	0.459	112.3
Among all (-)	-0.783	0.091	< 0.001	0.619	95.4
**Functional complexity (post de-trending)**					
Bact-Bact (+)	0.567	0.118	< 0.001	0.334	122.2
Fungi-Fungi (+)	0.514	0.123	< 0.001	0.274	126.4
Bact-Fungi (+)	0.481	0.128	< 0.001	0.234	129.0
Among all (+)	0.520	0.124	< 0.001	0.276	126.2
Bact-Bact (-)	-0.333	0.137	0.019	0.114	136.0
Fungi-Fungi (-)	-0.453	0.130	0.001	0.208	130.6
Bact-Fungi (-)	-0.263	0.145	0.077	0.067	138.4
Among all (-)	-0.301	0.139	0.036	0.092	137.1
